# Exploring the pharmacological potential of *Lepionurus sylvestris* blume: from folklore medicinal usage to modern drug development strategies using in vitro and in silico analyses

**DOI:** 10.1186/s12906-024-04567-2

**Published:** 2024-07-30

**Authors:** Laldinfeli Ralte, Hmingremhlua Sailo, Nachimathu Senthil Kumar, Y. Tunginba Singh

**Affiliations:** 1 Department of Botany, Mizoram University, Aizawl, Mizoram, 796004 India; 2 Department of Biotechnology, Mizoram University, Aizawl, Mizoram, 796004 India; 3 Department of Life Sciences (Botany), Manipur University, Imphal, Manipur, 795003 India

**Keywords:** LC-MS, Cytotoxicity, Antidiabetic potential, Molecular docking, MD simulation

## Abstract

**Background:**

*Lepionurus sylvestris* Blume has a long history of folklore medicinal usage against various ailments. However, studies on these plants were neglected particularly their pharmacological potential.

**Methods:**

The crude extract was identified using LC-MS analysis. In vitro assays were carried out to determine the properties of antioxidant, anti-microbial, and anti-cancer. Further, network pharmacology was proposed to evaluate the potential targets of the compounds against breast cancer and type II diabetes. Molecular docking and molecular dynamic simulation were used to determine the potential compounds for the drug formulation of diabetes.

**Results:**

Various bioactive compounds were identfied using LC-MS and Galiposin, Fujikinetin, Boeravinone B, 4-Deoxybryaquinone, and Norbaeocystin were described for the first time from the plant. Determination of antioxidant potential showed that the IC_50_ value of ABTS, DPPH, and phosphomolybdate was 24.33 µg/ml, 37.81 µg/ml, 60.35 µg/ml, and reducing power assays 1.185. The antibacterial activity against *Streptococcus pyogenes*, *Staphylococcus aureus*, *Pseudomonas aeruginosa*, and *Escherichia coli* was determined, and the minimum inhibition concentration (MIC) was found to be 5.3 mg/ml, 3.47 mg/ml, 3.33 mg/ml, and 2.7 mg/ml respectively, revealing the extracts as effective antibacterial agents. The IC50 values for the plant extract were determined to be 26 µg/ml, 30.52 µg/ml, and 24.39 µg/ml for HeLa, MCF-7, and K-562 cells, respectively, and the increasing concentration of the plant extract increased LDH release. Furthermore, the in silico network pharmacology, molecular docking which had the highest docking score for GAPDH and HIF-1 target proteins of -9.3 kcal/mol, and − 11.3 kcal/mol binding affinities, and molecular dynamic simulation analysis revealed the bioactive compound Boeravinone B present in the plant was significant for the treatment of various ailments.

**Conclusion:**

Based on our findings, plant extracts could be a promising option for developing new drug formulations.

**Supplementary Information:**

The online version contains supplementary material available at 10.1186/s12906-024-04567-2.

## Introduction

Traditional herbal medicines are gaining popularity since they are more natural, environmentally friendly, and free of adverse effects than modern synthetic and chemical drugs [1]. Due to the presence of various beneficial phytoconstituents in different plant parts, most medicinal plants have unique potential to treat and cure various diseases. [2]. Plant-derived medicines are commonly extracted from plant materials containing a complex blend of phytochemicals used to treat chronic and infectious diseases. [3]. It is crucial to develop effective screening methods when searching for new compounds and for quality control [4]. The extraction and characterization of various bioactive components from medicinal plants have led to the development of specific drugs with high activity potential [1, 5].

Medicinal plants are well known for their low toxicity and high concentrations of pharmaceutical chemicals, making them valuable as natural antioxidant agents [6]. The secondary metabolites present in plants, which possess antioxidant and antimicrobial properties, offer great potential for extracting beneficial phytochemical constituents. Compounds like phenols, flavonoids, and ascorbic acid can play a significant role in preserving the quality of plant products by effectively inhibiting oxidative reactions [7]. The free radical scavenging assay is commonly used to study the antioxidant properties of medicinal plants, as they may be involved in a variety of biological activities [8].

The increase in microbial resistance has prompted researchers to seek alternative solutions to antimicrobials. As a result, they have focused their efforts on natural products instead of chemical preservatives [7]. Numerous studies have highlighted the potential of bioactive compounds in medicinal plants as effective antimicrobial agents [9]. Furthermore, there is a growing need for innovative and less toxic anticancer treatments, and folkloric medicinal plants have emerged as a promising source of such agents [10]. Over 60% of anti-cancer medications are derived from plants, such as curcumin, cannabinoids, taxol, and paclitaxel [11]. Herbal remedies are gaining importance in the treatment of diabetic issues when compared to synthetic pharmaceuticals. A wide variety of medicinal plants are used to treat diabetes, and bioactive compounds with anti-diabetic properties have been identified in these plants. [12].

Due to their poor pharmacokinetic qualities, several medications have failed to reach the market, resulting in significant losses for pharmaceutical corporations [1]. Computer-assisted tools have evolved as sophisticated drug discovery methodologies that may be used to screen medicines derived from phytochemicals found in different medicinal plants [13]. Network pharmacology, which uses the cutting-edge idea of multicomponent-multitarget-multi-pathway, integrates various databases and investigates pharmacological mechanisms by utilizing bioinformatics, offering unique ways to research intricate herbal formulations [14]. Molecular docking is an effective and cost-effective method for creating and screening pharmaceuticals that provide information about drug-receptor interactions which can be used to anticipate how drug candidates will bind to their target proteins [15].

*Lepionurus sylvestris* Blume belongs to the family Opiliaceae and is the traditional lesser-known medicinal plant [16]. The plant is native to Mizoram, Yunnan, Bhutan, Assam, Indonesia, Laos, Malaysia, Myanmar, Nepal, Sikkim, Thailand, Vietnam [16]. The plants cure various ailments such as stomachache, cancer, diabetes, ulcers, etc. [16–18]. In Thailand and various parts of Southeast Asia, the plant is utilized as a body tonic, for addressing erectile dysfunction, as a diuretic, an aphrodisiac, and for the management of kidney stone diseases. [17]. As confirmed by the State Medicinal Plants Board in Mizoram, the plant has been officially listed as near threatened. Despite the ethnomedicinal uses, there is a lack of comprehensive study on these plants. While the ethnomedicinal uses have been documented, there is a need for further research on their pharmacological potential and bioactive components in the study area. As a result, the current study was designed to assess the in vitro antioxidant, antimicrobial, anticancer, and anti-diabetic potential of *L. sylvestris* leaf extract. Furthermore, the study also focused on the identification of the bioactive compounds using LC-MS. Following that, in silico network pharmacology and molecular docking were used to evaluate putative bioactive compounds for anticancer, and antidiabetic properties.

## Materials and methods

### Chemicals

All the reagents and solvents were of analytical grade, obtained from Hi-Media, Mumbai, India. ABTS (2,2- Azinobis-3-ethylbenzothiazoline-6-sulphonic acid disodium salt), DPPH (2,2-diphenyl-1-picrylhydrazyl), TPTZ (2,4,6 (2-pyridyl)-s-triazine), Ammonium molybdate A.R., Dimethyl Sulphoxide, Sodium acetate trihydrate ACS, Ferric chloride hexahydrate A.R., L-Ascorbic acid A.R., Acetic acid glacial A.R., Sodium carbonate ACS, Potassium persulphate A.R., were purchased from Hi-media, Mumbai, India.

### Plant material

The plant leaves were collected from Chhingchhip, Mizoram, Northeast India in October 2021. Plants were identified by Dr. Kh. Sandhyarani Devi, Taxonomist, Department of Botany, Mizoram University, and kept at the Department of Botany, Mizoram University, and voucher specimens were kept under reference number, *L. sylvestris* (MZ/BOT/122).

### Extraction preparation

The collected leaves were washed and dried at room temperature until they were completely dry and were ground into powder form. A 50 g of the powdered form was extracted with 500 ml of methanol using Soxhlet apparatus for 35 cycles at 35-40^o^C. Then, the extracts were concentrated in a water bath (50^o^C) to obtain crude extracts. Then, the concentrated extracts were kept at 4^0^C until further use.

### Antioxidant assay

#### DPPH free radical scavenging activity

The free radical scavenging potential of the extracts was determined by the method proposed by Brand-Williams et al. [19]. The percentage inhibition was measured as described earlier [7]. The IC_50_ value was determined by the graph plotting the inhibition percentage against the concentration.

### ABTS radical scavenging assay

The free radical scavenging activity was estimated by ABTS radical cation decolorization assay [20]. Briefly, ABTS solution (7 mM) was mixed with 2.45 mM potassium persulfate solution and maintained in the dark overnight to produce a dark-colored solution containing ABTS radical cations. The ABTS radical cation was diluted with 50% methanol before use in the test, resulting in an initial absorbance of approximately 0.700 at 734 nm at room temperature. Then, 100 µl extract was mixed with 3.900 ml of diluted ABTS solution and incubated at room temperature for 30 min. All the experiments were carried out in triplicate. The percentage inhibition was calculated using:

$$\eqalign{& ABTS\,scavenging\,effect\left( \% \right) = 100 \times (control\,absorbance \cr & \quad - sample\,absorbance)/(control\,absorbance) \cr}$$

#### Ferric-reducing antioxidant power (FRAP)

The antioxidant activity was determined by the spectrophotometric method proposed by Benzie & Strain [21]. Briefly,3.900 µl of the FRAP reagent (300 mM acetate buffer, 10 ml TPTZ, 40 mM HCl,20 mM FeCl_3_.6H_2_O with a ratio of 10:1:1) was mixed with 100 µl extract was incubated 30 min at room temperature. The absorbance was measured at 593 nm and the concentration was plotted against the standard concentration of ascorbic acid and expressed as mg of ascorbic acid equivalent per gram of sample.

### Phosphomolybdate assay

The total antioxidant capacity of the extract was determined by using the phosphomolybdate method [22] with slight modification previously done by Ralte et al. [6]. The total antioxidant capacity was calculated using:

$$\eqalign{& Antioxidant\,effect\left( \% \right) = 100 \times (control\,absorbance - \cr & \quad sample\,absorbance)/(control\,absorbance) \cr}$$

### Antimicrobial assay

#### Sample preparation

A 10 mg sample of crude extract was resuspended with 1 ml of methanol and different concentrations were obtained to evaluate the antimicrobial activity against the bacterial pathogens.

#### Bacterial strains

The bacterial pathogens used for the determination of antimicrobial activity were *Escherichia coli* (ATCC11229), *Streptococcus pyogenes* (ATCC19615), *Staphylococcus aureus* (ATCC23235), and *Pseudomonas aeruginosa* (ATCC9027).

#### Antibacterial screening

The initial antibacterial screening was determined using the agar well diffusion method [7]. The optical densities of the bacterial strains were adjusted to meet a 0.5 McFarland standard with 108 colony-forming units (cfu/ml) and distributed on agar plates. The 6 mm wells were created using a sterilized cork borer and 40 µl of plant extract at various concentrations was added. The antibiotic ampicillin (20 µg/disc) was used as a positive control and methanol was used as a negative control. A clear zone around the wells represents antibacterial activity. All the experiments were performed in triplicates.

#### Broth microdilution method

On a 96-well microtiter plate, the minimum inhibitory concentration (MIC) was determined against all the tested bacterial strains using the broth microdilution method [23]. Blanks were kept at different concentrations of plant extract, bacterial culture in methanol was employed as a negative control, and antibiotics ampicillin were used as a positive control. The plates were incubated at 37^o^C for 36 h and the absorbance was measured at 630 nm. All the experiments were performed in triplicates. The IC_50_ values were determined using a calibration curve generated by linear regression.

### Cytotoxicity assay

#### Cell lines and culture

Three cancer cell lines HeLa, MCF-7, and K-562 were used to screen the cytotoxic potential against the plant extracts. The cell lines were grown in DMEM supplemented with 10% inactivated Fetal Bovine Serum (FBS), penicillin (100 µg/ml), streptomycin (100 µg/ml), and amphotericin B (5 µg/ml) at 37^o^C in a humidified environment of 5% CO_2_. A trypsin solution was used to dissociate the cell (0.2% trypsin, 0.02% EDTA, 0.05% glucose in PBS). All studies were carried out in 96-well plates, and the stock cultures were produced in 25 cm^2^ culture flasks (Tarsons India Pvt. Ltd., Kolkata, India).

### MTT assay

The cytotoxic activity of the extracts was tested against the cell lines using an MTT reduction assay. Trypsinized HeLa, MCF-7, and K-562 cells monolayers were planted on 96-well plates at a cell density of 10 × 10^− 4^ cells per 100 µl media in each well and were incubated for 24 h at 37^o^C in a 5% CO_2_ environment. The cells were treated with the extract after the incubation at different concentrations (25, 50, 75, 100 µg/ml). For each sample, cells were incubated with 0.5% methanol as a blank, as well as untreated cells as a control, and incubated for 72 h. All the experiments were performed in triplicate. Then, the culture medium was removed from each well, and 20 µl of MTT (3-(4,5-dimethylthiazol)-2,5-diphenil-tetrazolium bromide (5 mg/ml in PBS) was added. DMSO was added to dissolve the MTT purple formazan after 4 h incubation and the absorbance was measured at 570 nm. The cell viability was calculated using the formula:

$$\eqalign{& Cell\,viability\left( \% \right) = 100 \times (sample\,absorbance - \cr & \quad sample\,blank)/\left( {sample{\mkern 1mu} control - sample\,blank} \right) \cr}$$

The half-maximal inhibitory concentrations (IC_50_) are the concentrations of the tested material required to inhibit 50% cell multiplication was also calculated using a non-linear regression curve in the Graph pad Prism statistical software.

### Lactate dehydrogenase (LDH) assay

Cell membrane rupture causes the discharge of the cytoplasmic enzyme lactate dehydrogenase. Damage to the plasma membrane is one of the signs of cellular death. The LDH cytotoxicity assay kit (Thermo Fisher Scientific Inc., Waltham, MA, USA) was used according to the manufacturer’s directions to test for this damage. To put it briefly, the cells were plated in a 96-well dish overnight, exposed to the cytotoxic extracts, and then incubated for 48 h. The wells of the highest LDH activity control received 10 µl of lysis buffer following 180 min of incubation at 37 °C. After that, 50 µl of the supernatant from each well was added to the reaction mixture. The mixture was then allowed to incubate for 30 min at room temperature in the dark. The standard used was sodium pyruvate. The LDH activity was then calculated using the following method after the absorbance at 420 nm was measured:

$$\eqalign{\% \,Cytotoxicity & = \,Absorbance\,at\,420\,nm\,of\,plant \cr & \quad extract\,treated\,sample \cr & \quad /Absorbance\,at\,420\,nm\,control \cr & \quad \times 100 - 100 \cr}$$

### LC-MS

The plant extract was LC-MS analyzed using an Agilent 1100 LC machine equipped with a degasser, binary pump, autosampler, and column heater. The column exit was attached to an Agilent MSD Ion Trap XCT mass spectrometer equipped with an ESI ion source and data analysis software was used to collect data and conduct mass spectrometric analysis. For the chromatography separation, a Zorbax 300 Extend-C-18 Column (2.1 150 mm) was used. The column was maintained at 95% solvent A (0.1% formic acid in water) and 5% solvent B (0.1% formic acid in acetonitrile) for 1 min, followed by an 11-minute step gradient from 5% B to 100% B, 4 min with 100% B, and 2 min with a linear gradient from 100% B to 5% B. The flow rate was 200 µl/min and the injection amount was 5 µl. Throughout all MS experiments, the capillary voltage was set to 3.5 kV, the drying temperature to 350 °C, the nebulizer pressure to 40 psi, and the drying gas flow to 10 µl/min for electrospray ionization with positive ion polarity. The ultra-scan setting, 26,000 m/z/s scan speed; fragmentation time, 30 ms with a 50 ms maximum accumulation period.

### Network Pharmacology for selected bioactive compound

#### Screening of potential targets

The 3D structure of the 18 active compounds was retrieved from the PubChem database (http://pubchem.ncbi.nlm.nih.gov/) and the target genes were downloaded from the Swiss target prediction Database (http://www.swisstargetprediction.ch). The GeneCards (http://www.GeneCards.org/) was used to obtain Breast cancer (BC), and type II Diabetes (DB) related targets. Jveen (http://jvenn.toulouse.inra.fr/) was used to identify the common targets between the compounds and diseases (Breast cancer and diabetes type II).

### Protein-protein Interaction (PPI) network construction

The String database (http://string-db.org) was used to create a PPI network to analyze the shared protein targets between the compounds and target diseases (BC & DB). The PPI interactions for *Homo sapiens* were evaluated with a confidence score greater than 0.9. To create the PPI network and retrieve hub genes, the TSV format of the PPI network was obtained and integrated into Cytoscape 3.9.1 software.

#### GO and KEGG pathway enrichment analysis

The Gene Ontology (GO) and Kyoto Encyclopedia of Genes and Genomes (KEGG) pathway analyses were executed to identify shared protein targets between compounds and BC and DB using ShinyGO 0.77 (http://bioinformatics.sdstate.edu/go/) and all the results were screened at *p* < 0.05. The top 20 genes were also visualized as bar plots and bubble plots using the bioinformatics database (www.bioinformatics.cn/en).

#### Component-target-pathway (C-T-P) network construction

Cytoscape 3.9.1 software was used to build a C-T-P network to analyze the intricate relationships between the compounds, target diseases (BC & DB), common targets, and pathways respectively.

#### Molecular docking

The 3D structure of HIF-1 (8HE3), and GAPDH (1DC3) were retrieved from the PDB database (https://www.rcsb.org/), dehydration, and dephosphorylation were done in Studio Discovery Visualizer software. The target protein and bioactive component binding affinity was calculated using AutoDock Vina. The docking results are regarded as reliable when the docking binding free energy is lower than − 5 kcal/mol. The ligand-protein interactions were visualized using Studio Discovery Visualizer.

### Molecular Dynamic (MD) simulation and principal component analysis (PCA)

The molecular dynamic and stability of top top-scoring BB_HIF-1 complex were studied using the GROMACS 2018.1 version. The system was created and tested using a 25 ns MD simulation to evaluate the stability of the BB_HIF-1 complex. In the presence of a solvent, the stability of the protein-ligand complex was investigated and a simple point charge model was used to solve the system in a cubic box. Further, the SWISSPARAM server (https://www.swissparam.ch/) was used to construct the ligand topology. The GROMACS 9653a6 force field was used to create protein topology [24] and the system was neutralized by the addition of 8 Na^+^ ions. The long-range electrostatic interactions were calculated using the Particle Mesh Ewald (PME) method [25]. For the sampling of the simulation trajectory, the time step was kept at 2 fs. Following energy minimization, the system was stabilized, and the volume, temperature, and pressure were then maintained via position restraint simulation under NVT and NPT conditions. The functions gmx rms, gmx rmsf, gmx gyrate, gmx hbond, and gmx sasa were used to compute the root-mean-square deviation (RMSD), root-mean-square-fluctuation (RMSF), the radius of gyration (Rg), hydrogen bond (H-bond), and solvent accessibility surface area (SASA). The gmx covar and gmx anaeig were used to perform the principal component analysis (PCA) and xmgrace was utilized to generate and visualize the graphs [26].

### Statistical analysis

Using IBM SPSS Statistics ver. 20, a statistical analysis of the data was performed. All the data were displayed as the averages ± SD of each triplicate, independently conducted analysis. One-way variance analysis was used to conduct the statistical analysis (ANOVA).

## Result and discussion

### Antioxidant potential

**Fig. 1:**
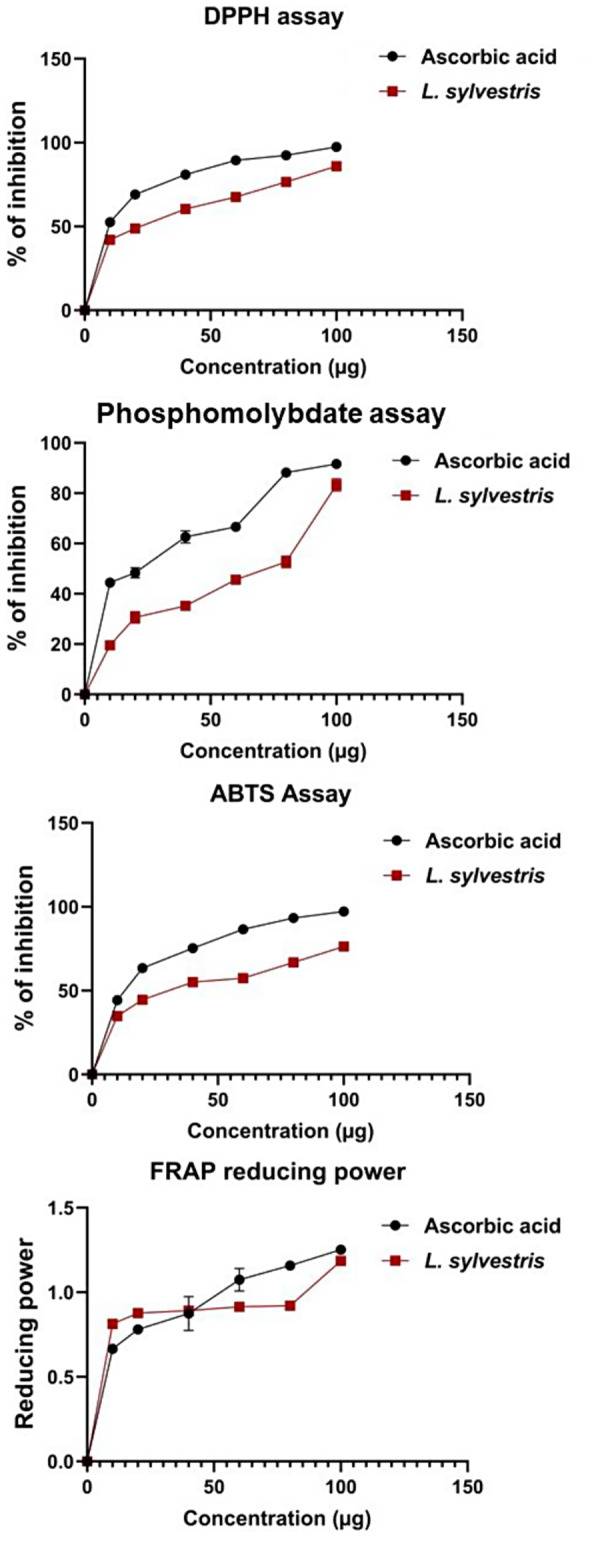
The percentage inhibition concentration of DPPH, ABTS, the total antioxidant capacity of phosphomolybdate, and the reducing power of FRAP assay

The antioxidant potential of the plant extracts was determined by using DPPH, ABTS, phosphomolybdate, and FRAP assays. The percentage inhibition, total antioxidant capacity, and reducing power are shown in Fig. 1. The IC_50_ values for DPPH, ABTS, and phosphomolybdate were also calculated and shown in Table 1. The IC_50_ values of DPPH, ABTS, and phosphomolybdate were found the highest in *L. sylvestris* at 24.33 µg/ml, 37.81 µg/ml, and 60.35 µg/ml respectively (Table 1). The antioxidant potentials of the compounds from the extracts are indicated by the reducing power assay. The reducing power of the extracts was verified in the present study over different concentrations (10–100 µg/ml). At the highest concentration (100 µg/ml), the FRAP-reducing power was found the highest in *L. sylvestris* (1.185) and was concentration-dependent (Fig. 1). The reducing power increases as the concentration increases and is comparable to the standard ascorbic acid. To the best of our knowledge, this is the first report on the antioxidant activity of DPPH, ABTS, phosphomolybdate, and FRAP in *L. sylvestris*.

**Table 1:** The antioxidant IC_50_ values of plant extracts using the different antioxidant assay

Sl No.	Species name	DPPH IC_50_ (µg/ml)	ABTS IC_50_ (µg/ml)	Phosphomolybdate IC_50_ (µg/ml)
1.	*L. sylvestris*	24.33	37.81	60.35
2.	Ascorbic acid	19.37	24.71	33.43

It is a well-known fact that free radicals play a significant role in causing clinical symptoms [27]. Antioxidants protect humans from various ailments by scavenging free radicals or by safeguarding cells through antioxidant defense mechanisms [7]. The antioxidant activity in plants cannot be determined by a single assay alone [28]. Therefore, we used four parameters (DPPH, ABTS, phosphomolybdate, FRAP) to support our research. The DPPH purple-colored solution bleaching method can be used to determine the electron-donating ability of natural compounds [29]. This method works by adding a radical species or antioxidant to the DPPH solution, which decolorizes it, indicating its scavenging ability. The plant extract in this study exhibited significantly higher inhibition percentages, indicating that the bioactive compounds in the extract can donate hydrogen ions to free radicals, thus neutralizing their potential harm. The ABTS radical scavenging assay involves the reaction of ABTS and potassium persulfate to produce a blue/green ABTS + chromophore. The results of this study show that the plant extract possesses notable ABTS scavenging properties. Based on the reduction of Mo (VI) to Mo (V) by the plant extracts, and the subsequent formation of green phosphate/Mo (V) compounds, the total antioxidant capacity of the plant extracts was determined using phosphomolybdate assay. Various flavonoids and polyphenols have been found to contribute significantly to the phosphomolybdate scavenging potential of medicinal plants [30]. The FRAP assay evaluates the antioxidant’s reducing capacity by converting a ferric tripyridyltriazine (Fe^3+^ TPTZ) complex to a colored ferrous tripyridyltriazine (Fe^2+^ TPTZ). The breakdown of the free radical chain is accomplished by donating a hydrogen atom. In our findings, as the concentration increased, the reducing power also increased and showed significant antioxidant potential.

### Antibacterial activity

**Fig. 2:**
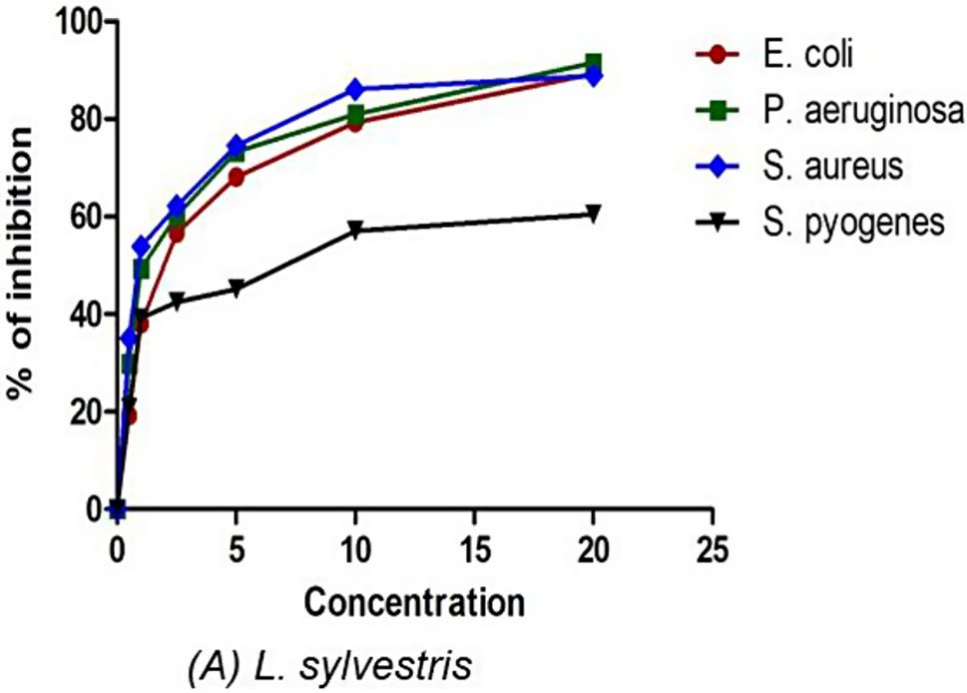
The percentage inhibition of the antibacterial against human pathogens

Table 2 shows the results of the antibacterial potential of crude methanolic leaf extract of *L. sylvestris*. The extract demonstrated significant antibacterial activity in the 9.1–18.2 mm range. In comparison to standard Ampicillin (30 g/mL), *L. sylvestris* showed a maximum inhibition zone against *E. coli* (18.2 mm). The inhibition concentration against the bacterial human pathogens is shown in Fig. 2. As the concentration increased the inhibition percentage also increased showing the concentration dependence of the extract.

**Table 2:** Antibacterial zone of inhibition of the plant extract against bacterial pathogens

Species name	Inhibition Zone (mm)				
	*S. pyogenes*	*S. aureus*	*P. aeruginosa*	*E. coli*	*Range*
*L. sylvestris*	9.1	11.6	15.4	18.2	High
Control					
Ampicillin	19	16	21	23	Very High

The minimum inhibition concentration (MIC) against the selected pathogens is shown in Table 3. The maximum inhibitory potential was shown in plant extract against *E. coli* (2.7) followed by *P. aeruginosa* (3.3), *S. aureus* (3.4), and *S. pyogenes* (5.3). The plant extract had a high MIC against pathogenic bacterial strains, indicating that the plant extract might be considered for the production of antibacterial drugs.

**Table 3:** The minimum inhibition concentration of *L. Sylvestris* extract against tested organisms

Sl. No	Species Name	MIC (mg/ml)			
		*S. pyogenes*	*S. aureus*	*P. aeruginosa*	*E. coli*
1.	*L. sylvestris*	5.3	3.4	3.3	2.7

The antibacterial properties of *L. sylvestris* indicate that it has significant potential in combating human pathogens such as *E. coli, P. aeruginosa, S. aureus*, and *S. pyogenes*, which are major causes of various infectious diseases. When compared to the well-known drug ampicillin (50 g/disc), the plant extract notably restrained gram-negative bacteria (18.2 mm) more than gram-positive bacteria (9.1 mm). The minimum inhibitory concentration (MIC) of the plant extract also demonstrated strong activity against the pathogens, suggesting that it could be utilized in the evaluation and development of new pharmaceutical drugs for controlling various bacterial infectious diseases. Plant phytochemicals are believed to possess antimicrobial properties, typically by impairing bacterial membrane function, thereby inhibiting or slowing bacterial growth [31]. The compound boeravinone B has been found to significantly inhibit NorA-mediated efflux in *S. aureus* [32]. Efflux pumps are primarily responsible for reducing the availability of drugs to block a specific target, which can decrease the concentration of antibacterial agents reaching the target and lead to novel resistance mechanisms [33, 34]. Therefore, the antibacterial properties demonstrated by the plant extract could be attributed to the presence of these compounds.

### Cytotoxicity assay

**Fig. 3:**
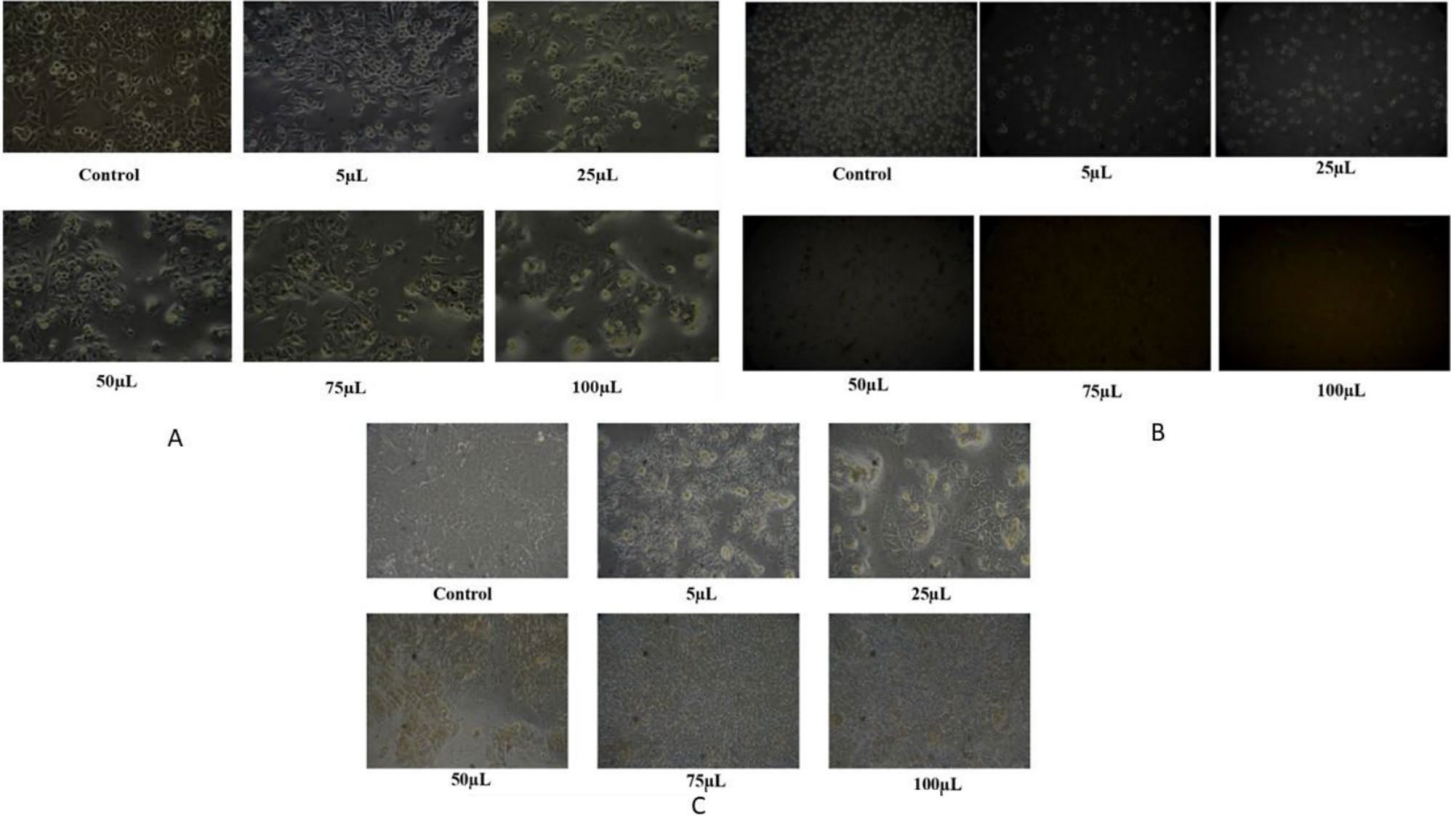
Changes in the morphology of cancer cells treated with the plant extract in cell culture media. A-HeLa; B-K562; C-MCF-7

**Fig. 4:**
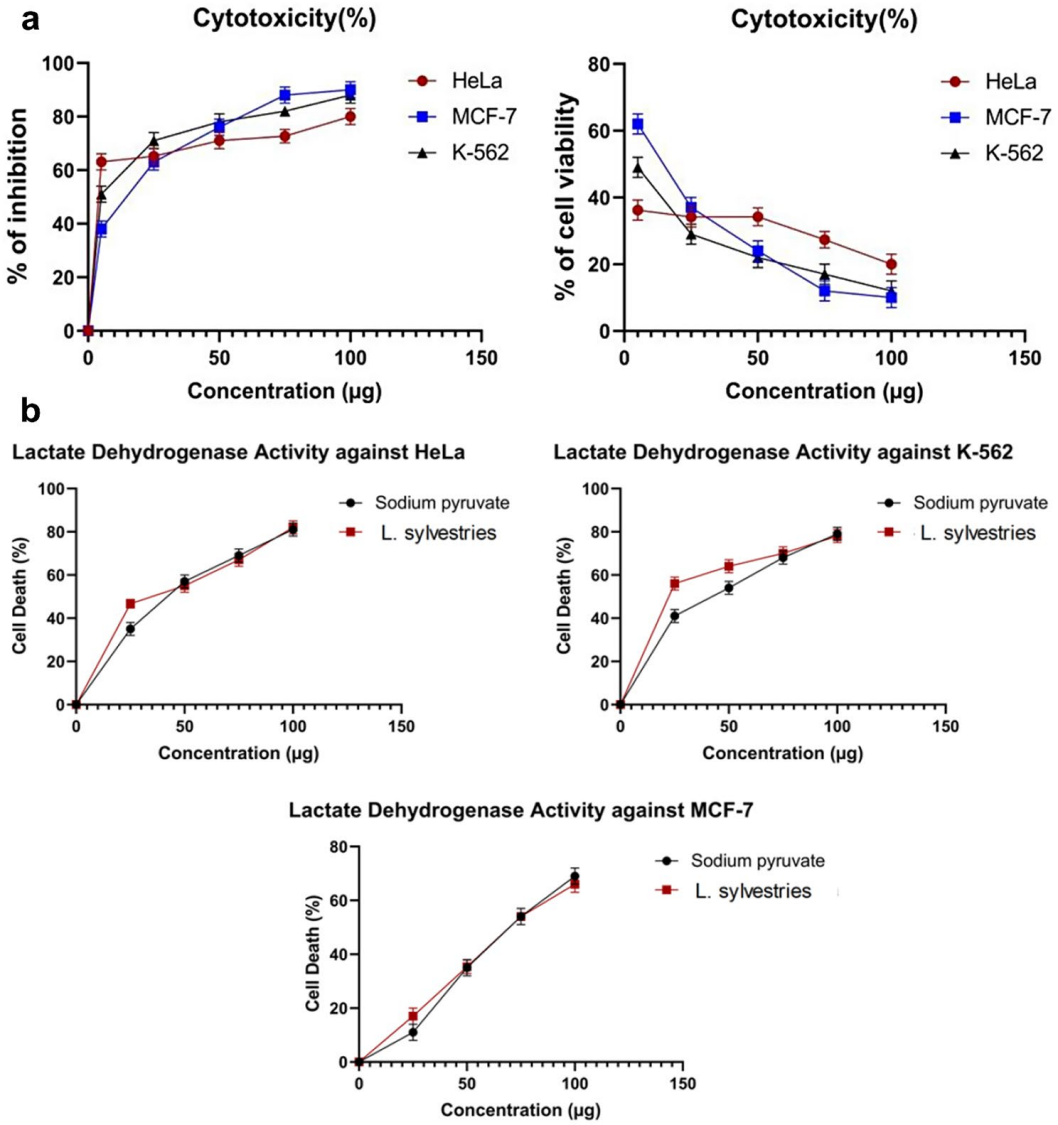
**a** The percentage inhibition and the percentage cell viability of *L. sylvestris* against the cancer cell lines (HeLa, MCF-7, and K-562). **b** The LDH level of *L. sylvestris* against

The MTT assay was used to assess the cytotoxic potential against the HeLa, MCF-7, and K-562 cancer cell lines (Fig. 3). The percentage inhibition (Fig. 4a) showed that the inhibition potential was concentration-dependent. The cell viability was plotted against the concentration, and it was shown that as the concentration increased, the viability of the cell decreased. The value of the IC50 for the inhibition of HeLa, MCF-7, and K-562 cells against the plant extract was found to be 26 µg/ml, 30.52 µg/ml, and 24.39 µg/ml respectively. The findings suggested that the extract might serve as a source of potential anticancer compounds.

A sign of injury or cell damage is a rise in the amount of lactate dehydrogenase (LDH), an enzyme that is necessary for all living cells. The cytotoxic potential of the plant extract was determined by measuring LDH release. The increasing concentration of the plant extract increased LDH release. Figure 4b displays the results of LDH, which support the findings of the MTT assay. In addition, the inhibition concentration (IC50) was also calculated (Table 4).

**Table 4:** The IC50 values of plant extract using LDH assay

Sl No.	Species Name	LDH IC_50_ (µg/ml)		
		HeLa	MCF-7	K-562
1.	*L. sylvestris*	50.49 ± 1.12	67.85 ± 0.71	44.7 ± 1.19
2.	Sodium pyruvate	52.04 ± 1.08	65.74 ± 1.21	52.16 ± 1.81

The plant extract was screened for cytotoxic potential using an MTT assay, which is a sensitive, quantitative, and reliable colorimetric assay that measures cell viability. The crude extract is typically deemed to have in vitro cytotoxic potential if the IC50 is 30–40 µg/ml [35]. The plant extract met this criterion and showed a strong cytotoxicity activity against the three cell lines suggesting that it could be a promising source as an anticancer agent. Due to the damaged cells being fully fragmented when exposed to a substance for an extended period, LDH assay is thought to be an accurate and reliable indicator of cytotoxicity [36]. When compared to the control in the current study, the LDH leakage in the cells treated with plant extract significantly increased.

### Liquid chromatographic-mass spectroscopy (LC-MS) analysis

**Fig. 5:**
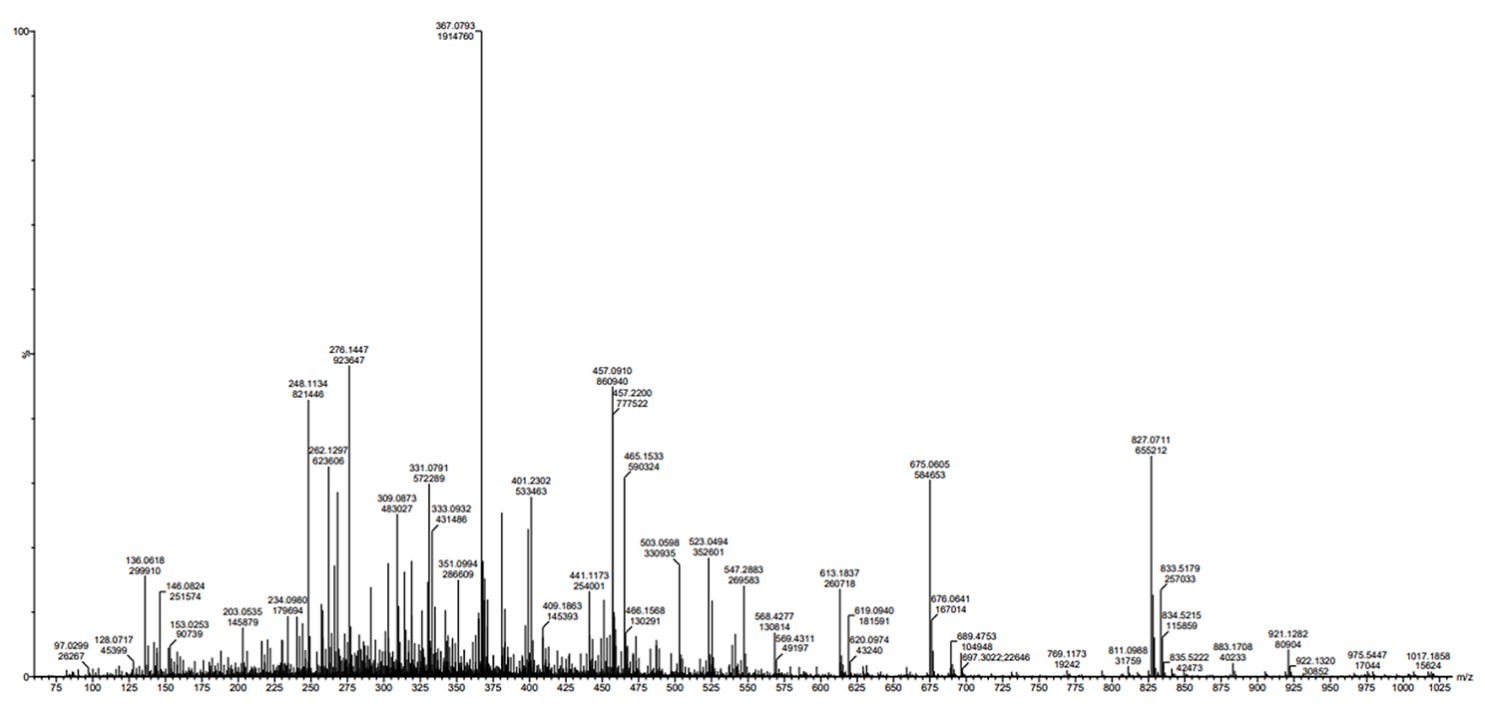
LC-MS chromatogram of *L. sylvestris* plant extract

The LC-MS profile of the plant extract revealed several peaks that each represented a distinct phytochemical component (Fig. 5). The analysis revealed the presence of 18 compounds such as Harmalol, Melinervin, Clorexolone, 3-Deazaguanylic acid, Fludiazepam, Deschloroetizolam, Desmethylindomethacin, Boeravinone B, Fujikinetin, Galiposin, Wairol, 4-Deoxybryaquinone, Prosogerin A, Quinoline, Carboxin sulfoxide, Phloroglucinol triacetate, Trisphaeridine, and Norbaeocystin.

The indole alkaloid compound harmalol possesses various pharmacological properties such as antimicrobial, antioxidant, and antiprotozoal activities [37]. The isoflavone fujikinetin, and prosogerin A compounds have been widely studied in vitro, in vivo, and in clinical trial trials due to their potential therapeutic benefits including anti-inflammatory, antioxidant, antidiabetic, and anti-cancer [38, 39]. The alkaloid compound quinoline has been shown to exhibit anti-microbial, and anticancer properties [40]. Phloroglucinol triacetate also has antimicrobial and antiviral properties [41]. Boervinone B has therapeutic potentials such as antioxidant, anticancer, and anti-inflammatory [42].

### Potential targets of active compounds and target diseases

**Fig. 6:**
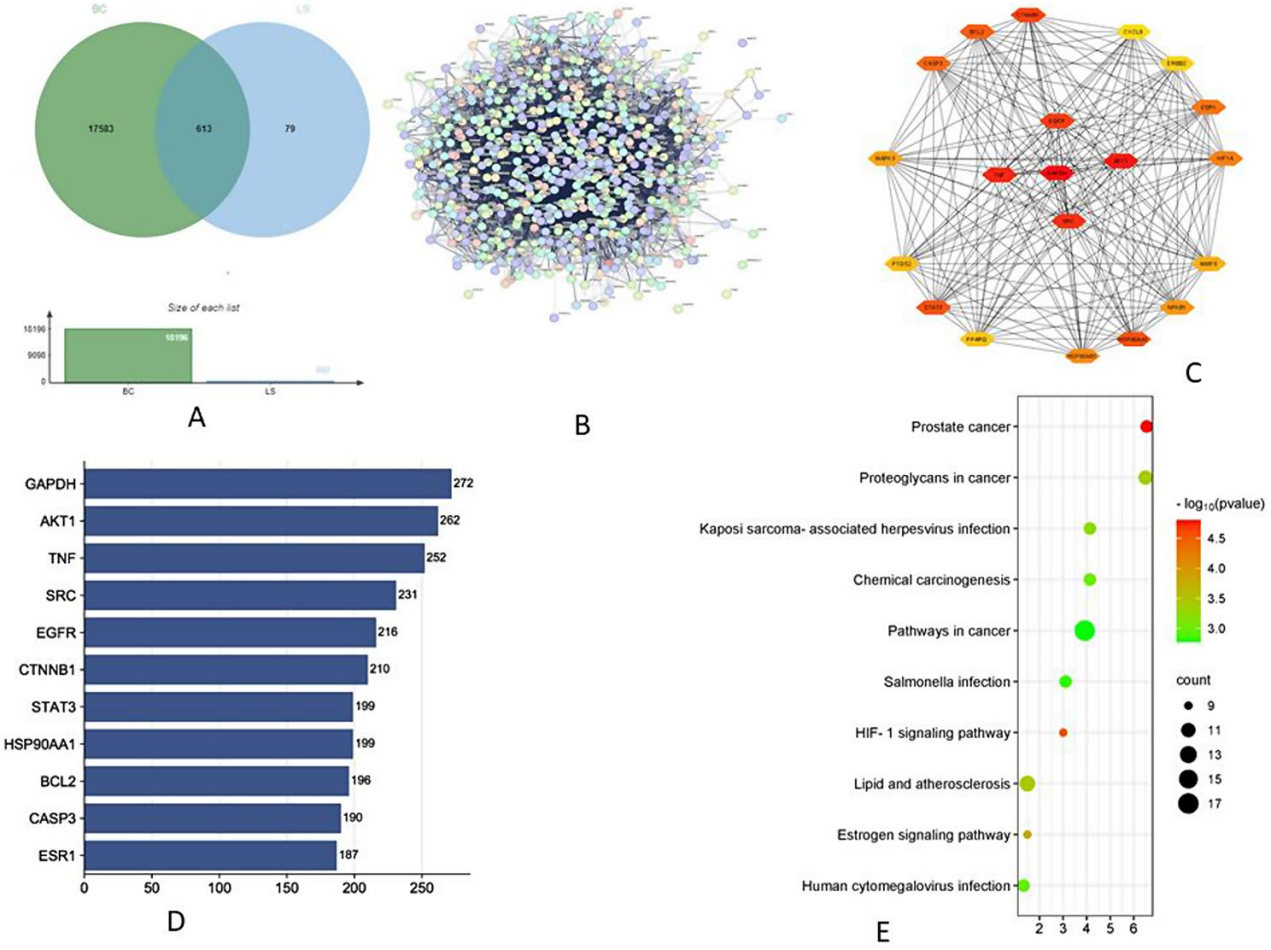
Analysis of network pharmacology. **A**- Venn diagram of active compounds of LS and breast cancer (BC) related targets; **B**- Protein-protein interaction network of BB in DB targets retrieved from STRING database; **C**- PPI network of common targets of BB and DB; **D**- Top 10 potential target genes and their gene counts; **E**- The best 10 enriched KEGG pathways of target genes in the treatment of BC

**Fig. 7:**
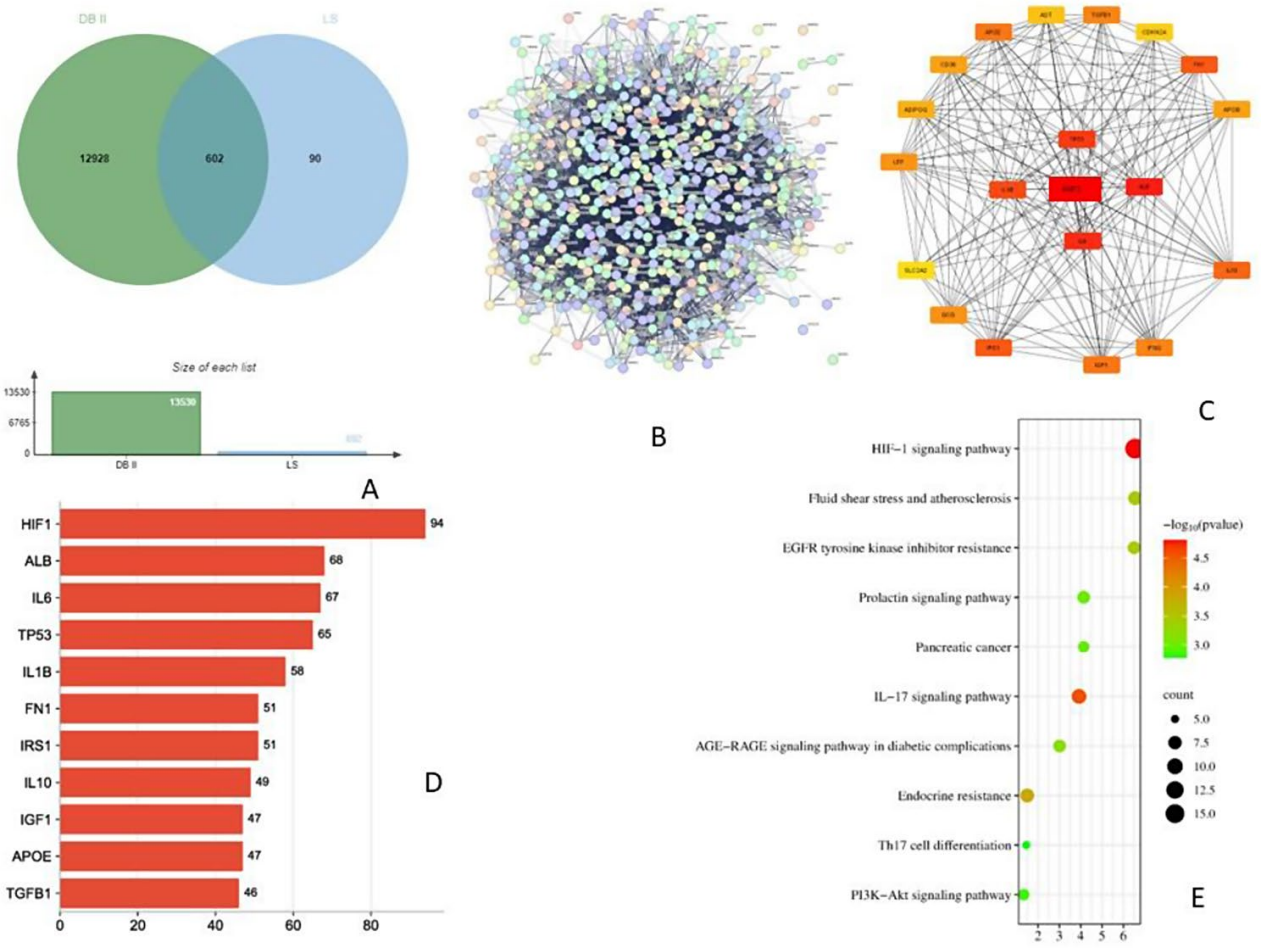
Network pharmacology - **A**- Venn diagram of active compounds of LS and type II Diabetes (DB) related targets; **B**- Protein-protein interaction network of BB in DB targets retrieved from STRING database; **C**- PPI network of common targets of BB and DB; **D**- Top 10 potential target genes and their gene counts; **E**- The best 10 enriched KEGG pathways of target genes in the treatment of DB

A total of 692 potential targets of active compounds of LS were obtained after deduplication in the UniProt database. Further, 18,196 BC-related and 13,530 DB-related targets were collected from the GeneCards database. By comparing the target genes of LS compounds with BC and DB, a total of 613 with BC, and 602 with DB genes respectively were found, suggesting that compounds of LS may exert the therapeutic effects of BC and DB with these identified target genes [Figs. 6A & 7A].

### Construction and analysis of target PPI network

**Fig. 8:**
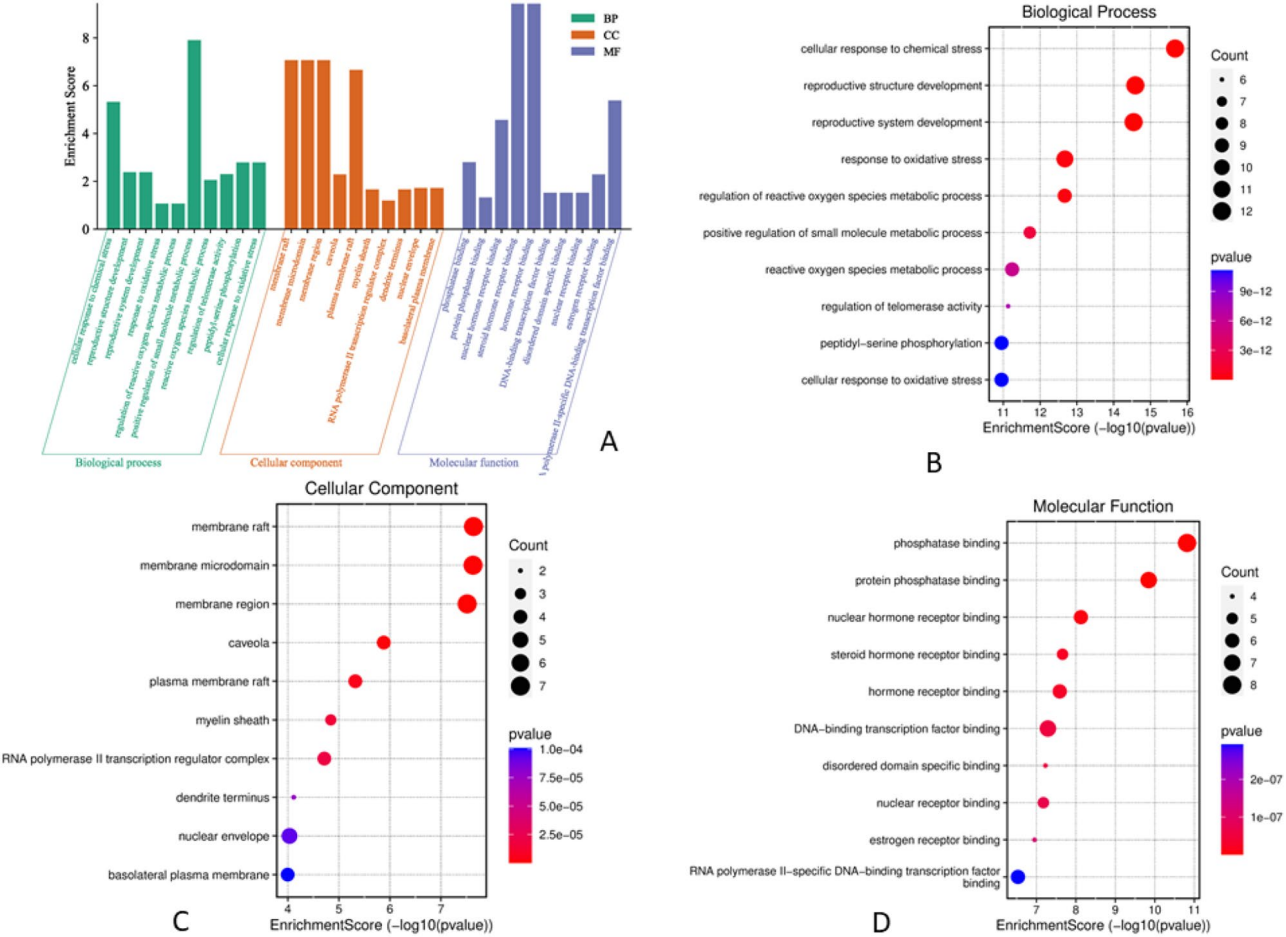
Analysis of GO function enrichment of LS compounds in the treatment of BC. **A** – The GO functional enrichment analysis of core targets for biological process (BP), cellular component (CC), and molecular function (MF); **B** – The top 10 GO enrichment in BP; **C** – The top 10 GO enrichment in CC; D – The top 10 GO enrichment in MF

A total of 613 and 603 potential targets for BC and DB were subjected to the STRING database for analysis (Figs. 6B and 7B). The target protein interaction was visualized in Cytoscape 3.9.1 software to construct a PPI network for BC and DB with active compounds (Fig. 6C & 7C). The interaction between the target proteins is shown in Figs. 7B and 8B which consisted of 612 and 601 nodes,11,493 and 11,542 edges, average node degree with values at 37.6 and 38.4, and local clustering co-efficient values of 0.446 and 0.448 for active compounds against BC and DB respectively. The topological analysis showed the presence of 20 major nodes with degree centrality (DC), closeness centrality (CLCE), and betweenness centrality (BC) were greater than the mean value (DC > 16, CLCE > 17.5, BC > 0.4). Furthermore, the highest five values were GAPDH, AKT1, TNF, SRC, and EGFR for LS-BC (Fig. 6D), while HIF-1, ALB, IL6, TP53, and IL1B were the top highest values for LS-DB (Fig. 7D).

### GO gene enrichment analysis and KEGG pathway annotation

**Fig. 9:**
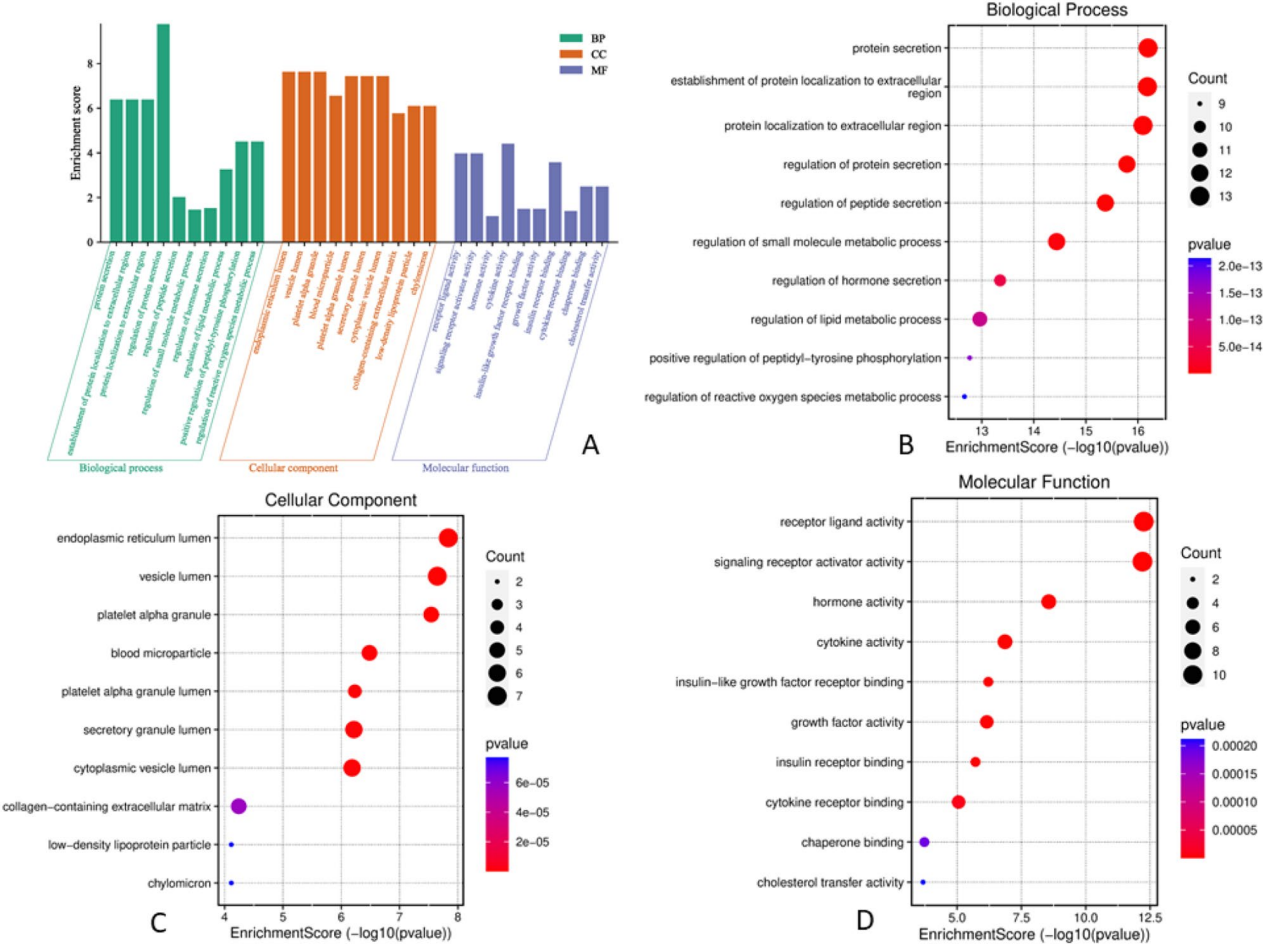
Analysis of GO function enrichment of LS compounds in the treatment of DB. **A** – The GO functional enrichment analysis of core targets for biological process (BP), cellular component (CC), and molecular function (MF); **B** – The top 10 GO enrichment in BP; **C** – The top 10 GO enrichment in CC; **D** – The top 10 GO enrichment in MF

The GO enrichment analysis of LS active compounds for the treatment of BC, and DB was performed using the ShinyGO database, to analyze the target genes for GO biological, GO molecular, cellular component, and KEGG pathway (Figs. 8 and 9). According to Benjamini-Hochberg’s false discovery rate, the GO term was limited to pV ≤ 0.005. The top 10 BP, CC, and MF functions are shown in bubble charts (Fig. 8BCD & 9BCD) for the treatment of BC and DB respectively. The targets of BP for the treatment of BC were enriched in response to chemical stress, reproductive structure, and system development, response to oxidative stress, metabolic process, regulation of telomerase activity, peptidyl-serine phosphorylation (Fig. 8B), CC analysis showed membrane raft, microdomain, plasma membrane raft, myelin sheath, RNA polymerase II transcription regulator, nuclear envelope (Fig. 8C), and MF analysis revealed including phosphatase binding, nuclear and steroid hormone receptor binding, DNA-binding transcription factor, estrogen receptor binding, and RNA polymerase II specific DNA-binding transcription factor binding (Fig. 8D). While for the treatment of DB, the BP targets include protein secretion, establishment of protein localization, regulation of protein, hormone and peptide secretion, regulation of small molecule, and lipid metabolic process, regulation of peptidyl-tyrosine phosphorylation (Fig. 9B), the CC analysis showed including endoplasmic reticulum, vesicle lumen, platelet microparticle, secretory granule lumen, collagen-containing extracellular matrix, and chylomicron (Fig. 9C), and the MF analysis results include receptor ligand activity, hormone and cytokine activity, insulin-like growth factor receptor binding, growth factor activity, insulin receptor binding, chaperone binding, and cholesterol transfer activity (Fig. 9D). A total of 139 signaling pathways involving prostate cancer, proteoglycans in cancer, pathways in cancer, HIF-1 signaling pathway etc., were the most significant enrichment pathway involved in LS compounds in the treatment of BC (Fig. 6E). Likewise, 96 signaling pathways were screened for LS compounds in the treatment of DB, and the top most significant pathways include HIF-1 signaling pathway, Fluid shear stress, and atherosclerosis, EGFR tyrosine inhibitor resistance, prolactin signaling pathway, IL-7 signaling pathway, AGE-RAGE signaling pathway in diabetic complications, PI3K-Akt signaling pathway, etc. (Fig. 7E).

### C-T-P network analysis

**Fig. 10:**
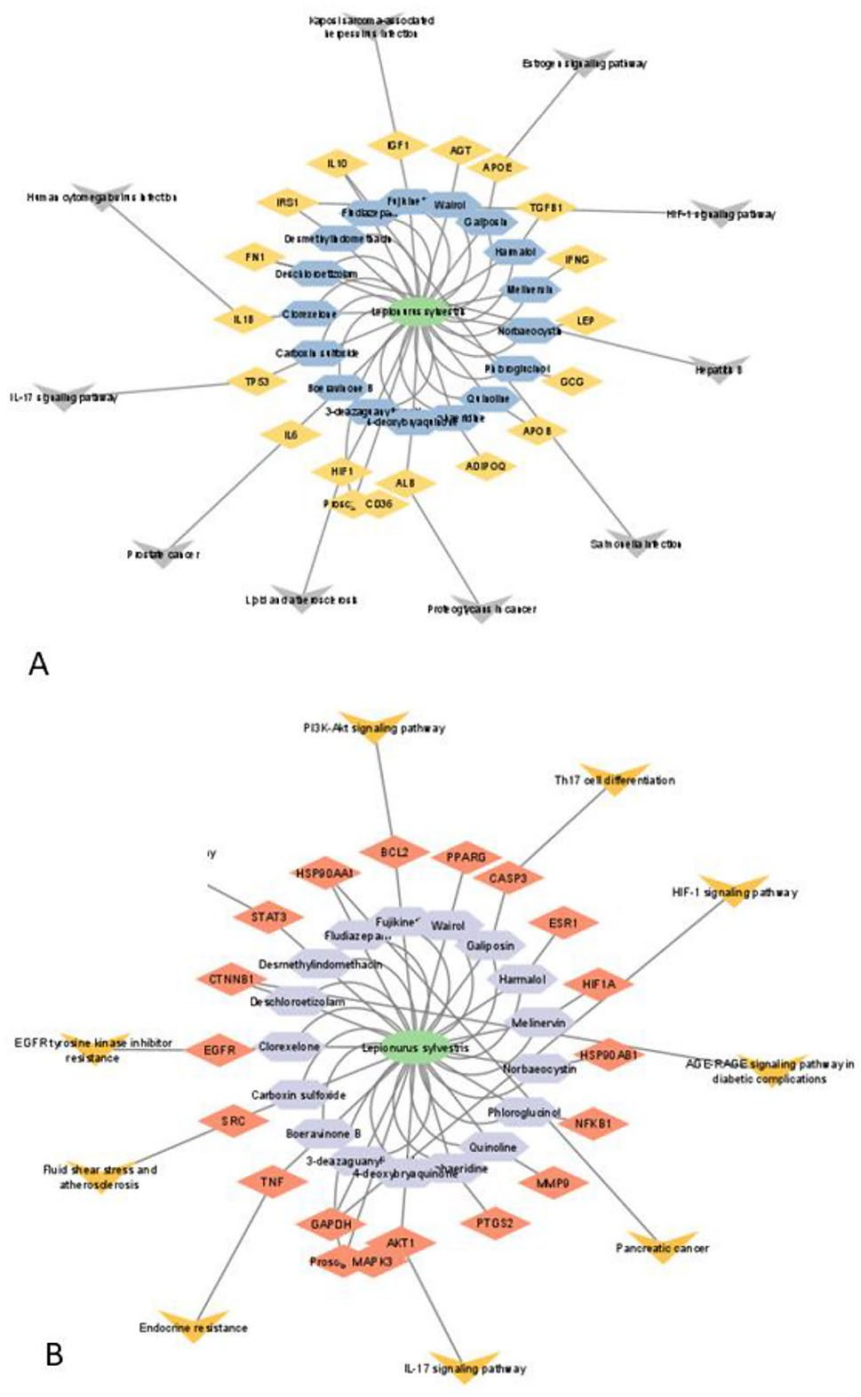
The component-target-pathways network, of LS compounds with their target genes, and signaling pathways. **A** – CTP network of LS with BC; **B** – CTP network of LS with DB

To create a C-T-P network, Cytoscape 3.9.1 software compiled and topologically examined the top 10 routes that corresponded to the targets. The network analysis suggests that one pathway, one drug, and one target may interact to affect different targets. This network diagram accurately depicted the synergistic interaction between various components, targets, and pathways used in the phytochemicals of LS treatment of BC and DB (Fig. 10A & B).

In the current landscape of drug development, a considerable focus lies on employing in silico techniques to assess the binding of compounds to established receptors. The utilization of high binding energies in this study adheres to the Lipinski rule of five parameters. Notably, our findings indicate that galiposin, fujikinetin, boeravinone B, and 4-deoxybryaquinone demonstrate significant drug-like properties.

In contrast to the traditional approach, network pharmacology focuses on understanding how medications relate to diseases through multi-targeted therapy, using a one drug, one target design. This method is innovative because it employs network analysis, connectedness, redundancy, and systems biology. Network pharmacology studies have been successful in identifying new targets and uncovering how unknown signaling pathways interact with compounds [44]. The NP method is a significant and promising technique for understanding disease mechanisms and identifying new bioactive compounds. It offers fresh insights into the relationship between treatment targets and the disease as a whole [45]. The current study revealed a unique network that provides a broad overview of the molecular processes of active compounds present in LS.

The homotetramer glyceraldehyde-3-phosphate dehydrogenase (GAPDH) is an important enzyme involved in producing energy in the cell and is also known to play a role in various physiological and pathophysiological processes, such as nuclear tRNA transport and apoptosis [46]. The regulation of GAPDH is influenced by several cancer-related factors. Studies have indicated that GAPDH is involved in tumor progression [47], and the expression of the GAPDH gene is increased in prostate, breast, lung, and cervical carcinomas [46]. Overexpression of GAPDH has been observed in breast cancer patients, with a 6.7-fold increase in those at stage IV [48].

Diabetes is associated with HIF-1, a transcription factor that plays a crucial role in maintaining oxygen balance [49]. HIF-1 expression indirectly affects renal oxygen metabolism [50]. Regulating the expression of target genes for downstream hypoxia response factors, as well as for cell energy metabolism, glucose metabolism, and apoptosis in hypoxic conditions, can help reduce cellular damage [51]. A GO enrichment study indicates that BB directly contributes to diabetes control. Furthermore, KEGG pathway analysis suggests that the HIF-1 signaling pathway may be significant in the identified network, supporting the hypothesis that BB could be used to treat diabetes. In the process of validating the targets, a docking study was carried out, which also involved evaluating the distance between the components and the target. This assessment directly revealed the relationship between their structure and activity. Furthermore, the results from network pharmacology demonstrated that BB therapy may effectively regulate the GAPDH and HIF-1 signaling pathways. These findings suggest that BC and DB could be effectively addressed using a comprehensive system with multi-component target disease interaction.

The structural and non-structural proteins of HIF-1 interact strongly with various phytochemicals found in plants, leading to changes in their structure. The diverse chemical and structural properties of these plant molecules serve as the basis for creating frameworks that have a strong attraction to the active site of HIF-1 receptors, thus paving the way for the discovery of new therapeutic targets.

### Molecular docking

**Fig. 11:**
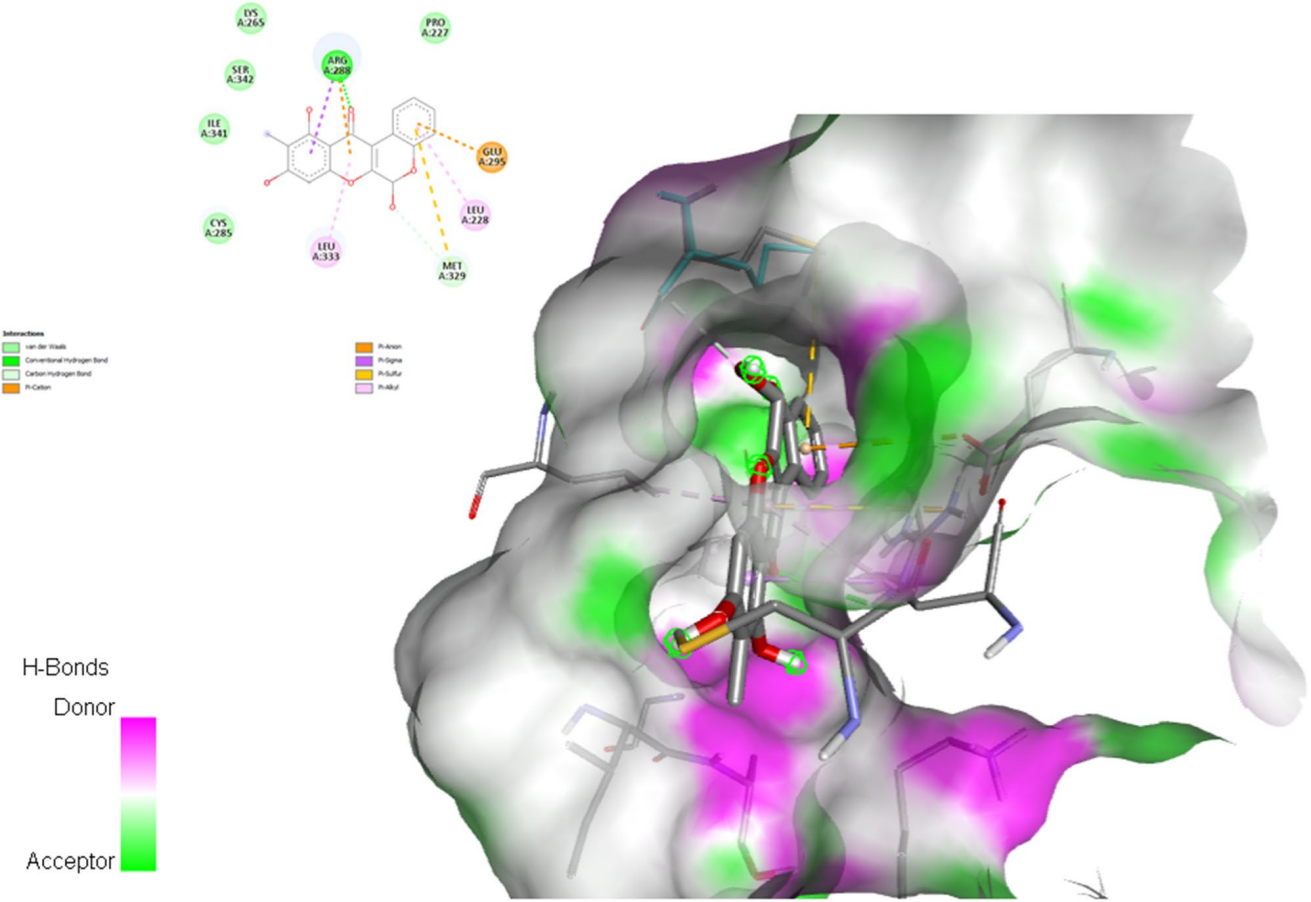
The protein-ligand interaction. Boeravinone B interacts with HIF-1 (8HE3

Based on the KEGG pathway and PPI network analysis, GAPDH and HIF-1 are potential targets for active compounds of LS to treat BC and DB, respectively. Subsequently, we conducted docking experiments using AutoDock Vina to assess the binding affinity of the compounds to these potential targets at the molecular level. Our analysis revealed significant binding affinities of the phytochemicals from LS (refer to Supplementary Table 1). Furthermore, we evaluated the potential pharmacokinetic properties of the compounds using the SwissADME database, confirming their potential drug-likeness properties (refer to Supplementary Table 2). Notably, Boeravinone B (BB) exhibited the highest docking score for GAPDH and HIF-1 target proteins, with binding energies of -9.3 kcal/mol and − 11.3 kcal/mol, respectively (see Fig. 11). Our findings strongly support BB’s favorable binding activity, validating the accuracy of our predictions in this study.

### MD simulation and PCA

**Fig. 12:**
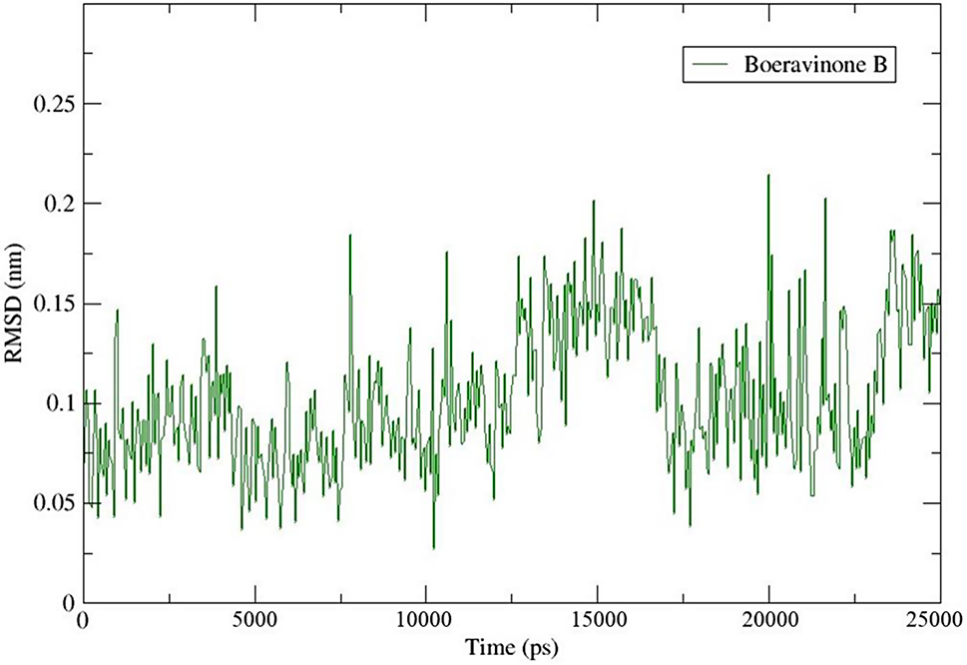
The RMSD for BB_HIF-1 complex with time in MD simulation

We investigated the stability of the BB compound binding to the HIF-1 target using MD simulation. We analyzed the RMSD plot, which shows the protein stability; higher RMSD values indicate more dramatic fluctuations. The system was set up for a 25 ns MD simulation, and the protein binding was stable (Fig. 12). The average RMSD value for the Cα backbone was 0.10 nm, indicating the stability of the protein-ligand complex.

**Fig. 13:**
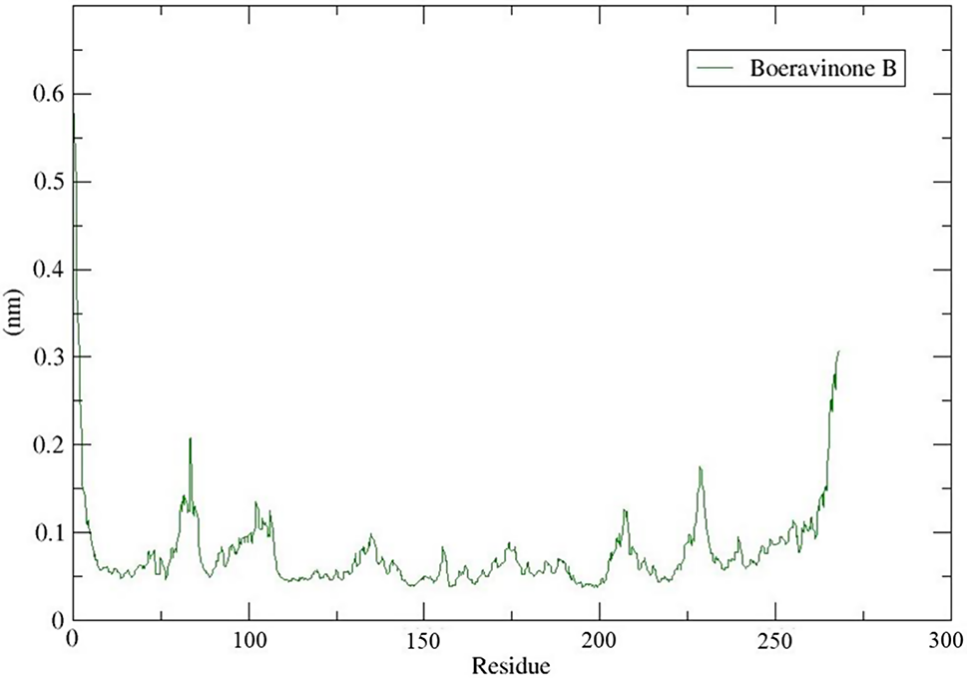
The RMSF plot of BB_HIF-1 complex

The conformational flexibility of the complex was examined by calculating the Root Mean Square Fluctuations (RMSF) of the Cα atom in the amino acid residue (see Fig. 13). The RMSF plot revealed the variation at the residue level, with an average RMSF value of 0.07 nm. The results indicated low conformational fluctuation, demonstrating the stability of the complex. The RMSF plot also suggested a stable binding of the phytochemical to the target protein.

**Fig. 14:**
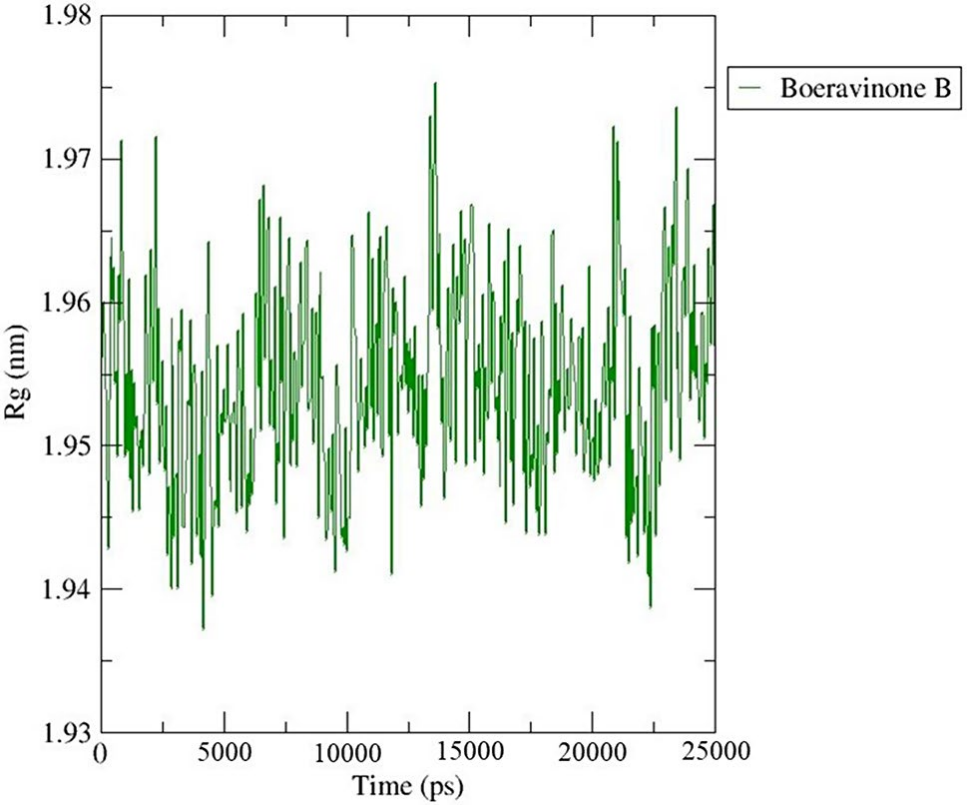
Radius of gyration (Rg) plot of BB_HIF-1 complex

The radius of gyration (Rg) of a structure indicates its compactness. When a system experiences minimal fluctuation, it becomes more compact and rigid, leading to stability throughout the simulation. To assess the compactness of all the complexes, we calculated the Rg values of the BB_HIF-1 complex (Fig. 14). The average Rg value of the complex was determined to be 1.67 nm, indicating a consistent fluctuation pattern. The complex exhibited stable fluctuation throughout the simulation period, demonstrating a high level of compactness and stability.

**Fig. 15:**
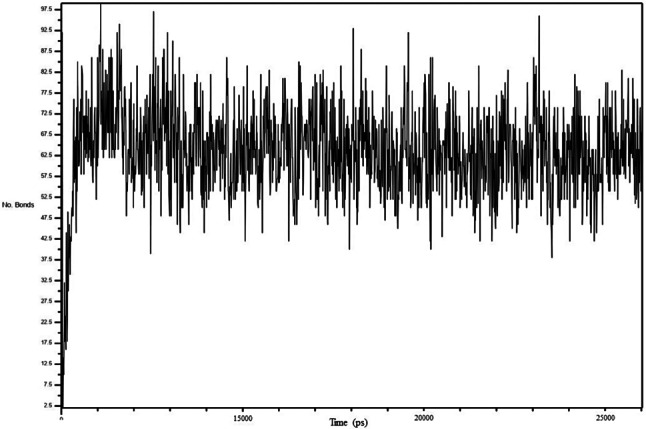
Analysis of numbers of hydrogen bonds of BB_HIF-1 complex

In molecular dynamics (MD) simulations for studying protein-ligand stability, analyzing hydrogen bonds is crucial. Hydrogen bonds are important interactions in biochemistry as they play a critical role in molecular recognition, structural stability, enzyme catalysis, drug partitioning, and permeability. The ability of drugs to form strong bonds with their biomolecular targets and their solubility both promote strong binding and selectivity, especially if they contain functional groups that can form hydrogen bonds [51, 52]. To assess the binding strength of the complex, we calculated the number of hydrogen bonds formed during the 25 nanosecond MD simulation (Fig. 15). The average number of hydrogen bonds was found to be 74, indicating that the complex was quite stable.

**Fig. 16:**
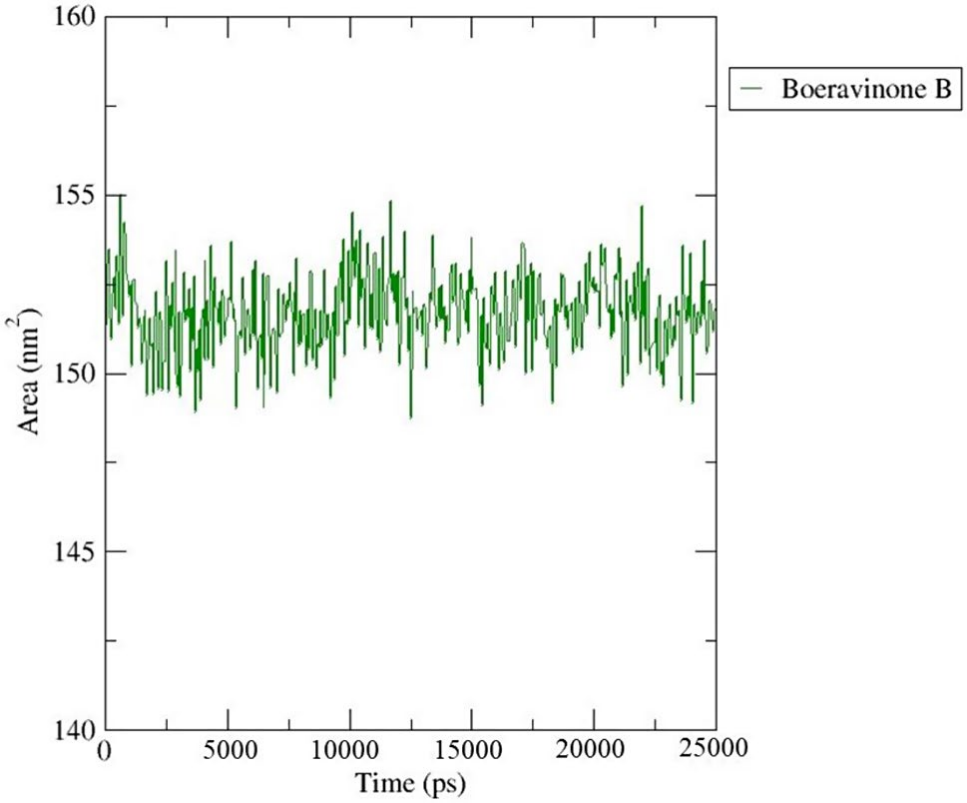
The solvent-accessible surface area of the complex

It has been suggested that the solvent-accessible surface area (SASA) plays a crucial role in studying molecular stability and folding. The SASA was used to calculate the expansion of protein volume in each system, allowing for a better understanding of the process (Fig. 16). Higher SASA values have the potential to indicate an increase in protein volume, with little variation expected over the simulation. Additionally, it is worth noting that the binding of a ligand could potentially alter the SASA and have a meaningful impact on the protein structure. The average SASA value was reported to be 151.69 nm2, and it was inferred from the observation that the binding of the BB could potentially reduce protein growth.

**Fig. 17:**
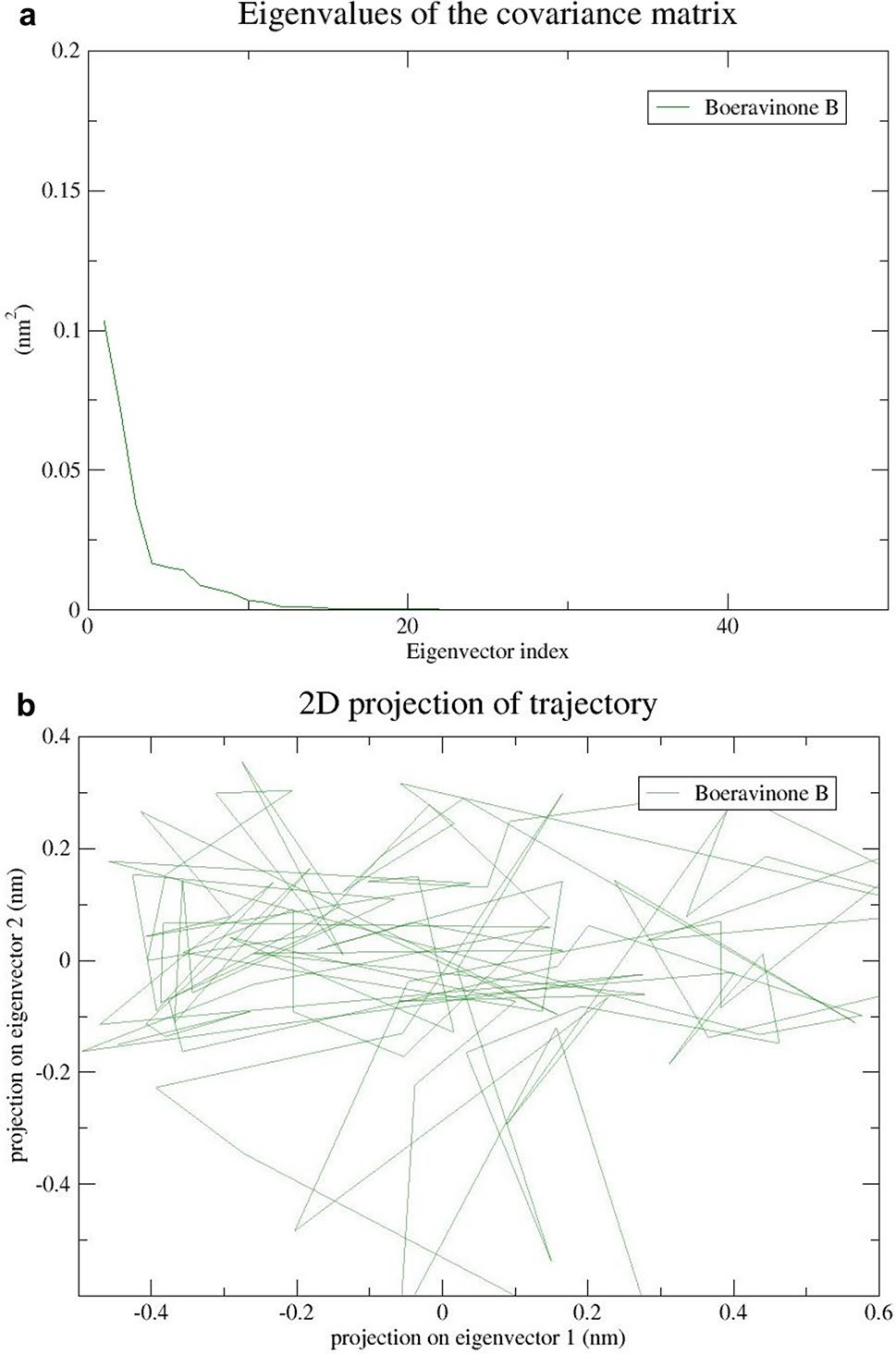
(**A**)Principal component plot of eigenvalue versus eigenvector index of the complex. (**B**) Principal component analysis. A 2D projection of protein movement

In this study, Principal Component Analysis (PCA) was used to analyze the movement of a protein called BB_HIF-1 Cα. The analysis utilized the gmx covar and gmx anaeig modules in GROMACS. By diagonalizing the covariance matrix, a flexible component was identified. The primary components of the covariance matrix are the eigenvectors. The eigenvalues and the matrix projected onto the eigenvector were graphed in Fig. 17A. The average eigenvalue of the complex was found to be 0.1nm^2^, indicating less correlated movements and suggesting that ligand binding promotes complex stabilization. To better illustrate the results, the first three eigenvectors were used to construct a 2D projection graph. The resulting plot (Fig. 17B) showed a stable cluster of the BB_HIF-1 complex in the phase space, giving us insight into the complex’s movements.

## Conclusion

The present study demonstrated that the plant extract under investigation displayed significant antioxidant, antimicrobial, and cytotoxic properties. Additionally, molecular docking and molecular dynamics simulation techniques were utilized in this study to screen phytochemical compounds with potential inhibitory effects against a diabetes-related target. The ligand required significant effort to dissociate from the target, indicating strong inhibitory potential. The compound also exhibited increased binding energies and formed stable complexes with the protein target. These findings suggest that Boeravinone B has substantial inhibitory potential against the target of diabetes and warrant further in vitro and in vivo studies. This supports the use of ethnopharmacological methods in selecting specific plant species for the discovery of new natural products. These findings may contribute to the exploration of underutilized medicinal plants as an important source of drug discovery.

### Electronic supplementary material

Below is the link to the electronic supplementary material.

Supplementary Material 1

## Data Availability

The datasets supporting the results of this article are included within the article and supplementary file. However, raw data used for analysis will be made available from the corresponding author on reasonable request.
