# Supplementary material for: Exploring the pharmacological potential of Lepionurus sylvestris blume: from folklore medicinal usage to modern drug development strategies using in vitro and in silico analyses
Source: BMC Complement Med Ther. 2024 Jul 30;24:289. doi: 10.1186/s12906-024-04567-2 (PMC11289938; doi:10.1186/s12906-024-04567-2)
Supplement: Supplementary file 1 — Supplementary Material 1 [file 12906_2024_4567_MOESM1_ESM.docx]

**Supplementary Table 1**. The docking score of the bioactive compounds against the target proteins.

| Sl No | Compounds | GAPDH (1DC3) (kcal/mol) | HIF-1 () (kcal/mol) |
| --- | --- | --- | --- |
|  | 3-deazaguanylic acid | -7.1 | -7.6 |
|  | 4-deoxybryaquinone | -6.9 | -7.9 |
|  | Boeravinone B | -9.3 | -11.3 |
|  | Carboxin sulfoxide | -6.2 | -6.5 |
|  | Clorexelone | -6.7 | -7.5 |
|  | Deschloroetizolam | -7.1 | -7.9 |
|  | Desmethylindomethacin | -7.1 | -7.8 |
|  | Fludiazepam | -7.1 | -7.8 |
|  | Fujikinetin | -7.1 | -7.7 |
|  | Galiposin | -7.4 | -8.3 |
|  | Harmalol | -6.2 | -7.1 |
|  | Melinervin | -7.4 | -8.5 |
|  | Norbaeocystin | -5.1 | -6.0 |
|  | Phloroglucinol | -5.7 | -5.9 |
|  | Prosogerin A | -7.1 | -8.0 |
|  | Quinoline | -6.4 | -7.3 |
|  | Trisphaeridine | -6.8 | -7.9 |
|  | Wairol | -7.0 | -8.5 |

**Supplementary Table 2**. The molecular properties, physicochemical and pharmacokinetics of the bioactive compounds

| Compounds | Molecular weight | Rotatable bonds | H-bond acceptor | H-bond donor atoms | Molecular refractivity | Polar surface area | Lipophilicity | Water solubility | Gastrointestinal absorption | Blood brain barrier permeation | P-glycoprotein substrate | Cyt P450 1A2 inhibitor | Cyt P450 2D6 inhibitor | Cyt P450 3A4 inhibitor | Druglikeness (Lipinski rule) |
| --- | --- | --- | --- | --- | --- | --- | --- | --- | --- | --- | --- | --- | --- | --- | --- |
| 3-deazaguanylic acid | 362.23 g/mol | 4 | 9 | 6 | 78.61 | 202.96Å^2^ | -2.63 | Soluble | Low | No | No | No | No | No | No |
| 4-deoxybryaquinone | 312.27 g/mol | 2 | 6 | 0 | 79.44 | 74.97Å^2^ | 1.92 | Soluble | High | Yes | No | Yes | No | Yes | Yes |
| Boeravinone B | 312.27 g/mol | 0 | 6 | 3 | 82.63 | 100.13Å^2^ | 2.12 | Soluble | High | No | No | Yes | Yes | Yes | Yes |
| Carboxin sulfoxide | 251.30 g/mol | 3 | 3 | 1 | 66.76 | 74.61Å^2^ | 1.31 | Soluble | High | Yes | No | No | No | No | Yes |
| Clorexelone | 328.81 g/mol | 2 | 4 | 1 | 84.22 | 88.85Å^2^ | 1.93 | Soluble | High | No | Yes | Yes | No | No | Yes |
| Deschloroetizolam | 308.4 g/mol | 2 | 3 | 0 | 93.29 | 71.31Å^2^ | 3.05 | Soluble | High | Yes | Yes | Yes | No | Yes | Yes |
| Desmethylindomethacin | 343.76 g/mol | 4 | 4 | 2 | 91.65 | 79.53Å^2^ | 3.21 | Moderately soluble | High | No | No | Yes | No | No | Yes |
| Fludiazepam | 302.73 g/mol | 1 | 3 | 0 | 87.91 | 32.67Å^2^ | 3.18 | Soluble | High | Yes | No | Yes | Yes | No | Yes |
| Fujikinetin | 312.27 g/mol | 2 | 6 | 1 | 82.50 | 78.13Å^2^ | 2.55 | Soluble | High | Yes | No | Yes | Yes | Yes | Yes |
| Galiposin | 312.27 g/mol | 3 | 6 | 1 | 79.95 | 74.22Å^2^ | 2.57 | Soluble | High | Yes | No | Yes | Yes | Yes | Yes |
| Harmalol | 200.24 g/mol | 0 | 2 | 2 | 64.76 | 48.38Å^2^ | 1.99 | Soluble | High | Yes | Yes | Yes | No | No | Yes |
| Melinervin | 374.3 g/mol | 3 | 9 | 3 | 93.04 | 127.82Å^2^ | 1.96 | Moderately soluble | High | No | No | Yes | No | Yes | Yes |
| Norbaeocystin | 256.19 g/mol | 4 | 5 | 4 | 63.45 | 118.38Å^2^ | -0.14 | Soluble | High | No | No | No | No | No | Yes |
| Phloroglucinol | 126.11 g/mol | 0 | 3 | 3 | 32.51 | 60.69Å^2^ | 0.45 | Soluble | High | Yes | No | No | No | Yes | Yes |
| Prosogerin A | 312.27 g/mol | 2 | 6 | 1 | 82.50 | 78.13Å^2^ | 2.55 | Soluble | High | Yes | No | Yes | Yes | Yes | Yes |
| Quinoline | 129.16 g/mol | 0 | 1 | 0 | 41.74 | 12.89Å^2^ | 2.08 | Soluble | High | Yes | No | Yes | No | No | Yes |
| Trisphaeridine | 223.23 g/mol | 0 | 3 | 0 | 65.31 | 31.35Å^2^ | 2.89 | Soluble | High | Yes | Yes | Yes | Yes | Yes | Yes |
| Wairol | 312.27 g/mol | 2 | 6 | 1 | 84.77 | 82.04Å^2^ | 2.85 | Moderately Soluble | High | No | No | Yes | Yes | Yes | Yes |
